# Living with hypertrophic cardiomyopathy and an implantable defibrillator

**DOI:** 10.1186/s12872-017-0553-y

**Published:** 2017-05-10

**Authors:** Peter Magnusson, Jessica Jonsson, Stellan Mörner, Lennart Fredriksson

**Affiliations:** 1 Cardiology Research Unit, Department of Medicine, Karolinska Institutet, Karolinska University Hospital/Solna, SE-171 76 Stockholm, Sweden; 2 Centre for Research and Development, Uppsala University/Region Gävleborg, SE-801 87 Gävle, Sweden; 3 Department of Public Health and Clinical Medicine, Umeå University, SE-90187 Umeå, Sweden

**Keywords:** Content analysis, Hermeneutics, Hypertrophic Cardiomyopathy, Implantable cardioverter defibrillator, Interview, Qualitative

## Abstract

**Background:**

ICDs efficiently terminate life-threatening arrhythmias, but complications occur during long-term follow-up. Patients’ own perspective is largely unknown. The aim of the study was to describe experiences of hypertrophic cardiomyopathy (HCM) patients with implantable defibrillators (ICDs).

**Methods:**

We analyzed 26 Swedish patient interviews using hermeneutics and latent content analysis.

**Results:**

Patients (aged 27–76 years) were limited by HCM especially if it deteriorates into heart failure. The ICD implies safety, gratitude, and is accepted as a part of the body even when inappropriate ICD shocks are encountered. Nobody regretted the implant. Both the disease and the ICD affected professional life and leisure time activities, especially at younger ages. Family support was usually strong, but sometimes resulted in overprotection, whereas health care focused on medical issues. Despite limitations, patients adapted, accepted, and managed challenges.

**Conclusion:**

HCM patients with ICDs reported good spirit and hope even though they had to adapt and accept limitations over time.

## Background

The hypertrophic cardiomyopathy (HCM) phenotype is diagnosed when the left ventricular wall is thicker than 15 mm without any other explanation [1]. HCM prevalence is approximately 1:500 in the general population but 1:300 if genotypes are also included [2, 3]. A mutation is found in more than half of the cases and can be used for screening of family members [1]. Genetic screening is sometimes the way to diagnosis but an abnormal ECG or a murmur may lead to evaluation with echocardiography. Symptoms like shortness of breath, chest pain, tiredness, dizziness, or syncope are unspecific. Disease progression varies greatly, and atrial fibrillation and end-stage heart failure with low ejection fraction imply a worse prognosis [4, 5]. Sudden cardiac death (SCD) is difficult to predict but can be effectively prevented by inserting an implantable cardioverter defibrillator (ICD) [6–8] which terminates ventricular tachycardia or ventricular fibrillation by anti-tachycardia pacing or shock discharge. In survivors of cardiac arrest or ventricular tachycardia with hemodynamic compromise, implanting an ICD is standard practice defined as secondary prevention [1, 9]. For primary prevention patients, the decision to implant an ICD requires careful clinical judgement based on risk markers [1, 9]. Current guidelines also take into account the lifelong risk of complications and the impact of an ICD on lifestyle, socioeconomic status and psychological health [1]. However, knowledge gained from several studies on general ICD populations cannot be generalized, because HCM patients are generally younger, have an extended life expectancy, suffer from other symptoms, and have a genetic disease. Taken globally, these conditions may affect an individual’s lifestyle, working life, family structure, leisure pursuits, and overall attitudes about life. Subasic concluded from 15 selected patients that living with HCM altered identity and generated fear and uncertainty [10]. Nevertheless, no previous study specifically addressed unselected HCM patients with ICDs. The aim of this study was to explore the individual experience of patients who had HCM and ICDs.

## Methods

The methodology is inspired by hermeneutics and also by latent content analysis [11–13]. The most fundamental structure of understanding within hermeneutics is the hermeneutic circle. In this study it is visible ‘both as a movement between tradition and the movement of the interpreter.’ [14] This presupposes a consciousness of the fact that the researchers are situated within a tradition which imply structures of pre-understanding, but also are capable by reflection to alter this understanding. The circle can also be envisioned as a movement between the parts and the whole when interpreting a single interview and also as parts and a whole when several interviews are translated into each other.

### Inclusion criteria, setting, procedures, and ethics

To cover essential aspects of the heterogeneous disease HCM we predefined the following maximum variation sampling variables: sex, age, time since diagnosis, primary/secondary indication of ICD, and a history of appropriate or inappropriate shock (Table 1). All patients, aged ≥18 years with at least 2 years history of a transvenous ICD due to HCM, were identified from the Swedish ICD Registry which has a complete coverage of all implants [15]. Patients with a postal address in the Region Gävleborg or Umeå University hospital and their affiliated hospitals were recruited.

**Table 1:** Characteristics of 26 interviewed HCM patients with history of ICD

Sex, age	Civic status	Child	Indication	ICD duration	ICD shock	Diagnosis	NYHA
M, 27	Cohabitate	0	primary	4.9	no	9	I
F, 32	Cohabitate	2	primary	2.4	no	17	II
F, 33	Cohabitate	2	secondary	6.3	no	14	I
M, 37	Divorced	1	primary	3.0	no	8	II
M, 42	Married	2	secondary	8.4	inappropriate	9	I
M, 48	Divorced	2	primary	4.8	no	30	I
M,49	Cohabitate	0	secondary	6.9	appropriate	7	II
F, 54	Cohabitate	1	primary	10.9	inappropriate	20	II
M,55	Single	0	secondary	8.0	appropriate	37	IIIB
M, 59	Married	2	primary	3.8	inappropriate	4	I
M, 59	Cohabitate	2	primary	1.0	no	32	IV/I^a^
F, 60	Married	3	secondary	10.9	inappropriate	20	I
M, 61	Married	2	primary	8.9	no	18	I
M, 61	Divorced	5	primary	11.3	inappropriate	45	IIIB
M, 63	Married	2	primary	16.6	appropriate	17	I
M, 64	Cohabitate	2	primary	3.0	no	4	I
F, 65	Single	1	primary	5.3	inappropriate	8	I
M, 65	Married	1	primary	5.1	no	42	I
M, 65	Married	2	primary	7.6	no	9	I
M, 67	Married	2	primary	3.2	no	4	II
F, 68	Married	4	primary	7.8	no	7	I
M, 69	Married	1	primary	4.5	no	5	I
M, 72	Divorced	7	secondary	3.5	no	20	II
F, 75	Divorced	0	primary	11.2	inappropriate	15	I
F, 75	Married	1	primary	9.8	inappropriate	14	IIIA
F, 76	Single	2	primary	5.2	no	7	I

*M* Male, *F* Female, *NYHA* New York Heart Association, *ICD* Implantable cardioverter defibrillator, *HCM* Hypertrophic cardiomyopathy

*ICD duration* refers to time (years) with an ICD and *Diagnosis* time (years) since first known diagnosis of HCM

^a^heart transplant due to NYHA IV, at the time of interview NYHA I

Medical data were validated using medical records (PM, SM). The patients were contacted by phone by the investigators (PM or SM) and scheduled for an appointment with the interviewer (JJ). All patients came to the appointment, consented, and were subsequently interviewed (sample size, *n* = 26) between February and June 2015 either at the research department, outpatient clinic, or at home. The study was approved by the Regional Ethical committee in Uppsala (Dnr 2015/060). References to patients’ identities have been omitted due to confidentiality.

### The interviews

Following information about the study, written consent, questions about age and habitual status, an open-ended communication was initiated. An interview guide was constructed based on the aim of the study using a narrative, explorative approach. Based on the researcher’s clinical experience and literature search in the field, the topics were developed. The topics covered wide areas of life experiences (see Appendix 1). The guide provided open-ended questions and also specific questions on each topic. This ensured that all relevant topics were addressed in each interview. The guide served as a narrative framework to achieve structure but encouraged the participants to speak freely and raise issues of concern to them. At the end of the interview, the guide was used to check for completeness. After each interview, a contact with their clinician was arranged or supported if the patient desired. Interviews were digitally audio-recorded and transcribed verbatim. The mean duration of interviews was 135 min (in total 58.6 h; range 81–210 min).

### Analysis and interpretation of interviews

The analysis and interpretation of the interviews was guided both by an awareness of the movement in the hermeneutic circles, but also by latent content analysis as a means to condense and code the text (Table 2). All interviews were read as a holistic narrative and discussed among the researchers. Through repeated reading, the interviews were condensed into meaning units, according to the aim of the study. These condensed meaning units were shortened and labelled with a code. The text was decontextualized, meaning that codes were read across interviews. Preliminary themes were interpreted and reflected upon, moving between parts and the whole. The process resulted in ten narrative themes and five theoretical themes, together ‘unfolding a world in front of the text.’ [16] Finally, all interviews were read again to verify the accuracy of interpretations and the reported themes (Table 2).

**Table 2:** Examples of content analysis and hermeneutic interpretation of narratives of hypertrophic cardiomyopathy and ICD

Condensed meaning unit	Code	Narrative themes	Theoretical themes
*It is constantly in the back of my mind. And it has surely become more visible for me because I received this device. It is therefore something that reminds me every day. Earlier – before I received the device- it was a reminder when I was called in for a check-up. At that time it was longer between the occasions…but it is nothing…yeah…it is part of the person I am.*	ICD is a reminder of disease. ICD is internalized and considered as part of the patient.	Implant decision, surgery, and wearing an ICD	Awareness
			Acceptance
Does the ICD affect your health? *Not at all. I used to let people touch it, feel what I have under my skin! I think it is kind of amusing.*	Appraisal focused strategy using humor but also denial.	ICD provides assurance	Adaptation
*I am comfortable with it. It is such a security and I am so grateful for getting the opportunity to do it* [ICD implant].	ICD implies safety and gratefulness.	ICD knowledge and worries	Gratitude
*It* [the ICD] *is almost on the outside, it seems to me. One can feel the wires here…but that is the only thing. One has to be careful not the get a hit here when playing with the grandchildren.*	Local problems superficial device system.	Implant decision, surgery, and wearing an ICD	Awareness
			Adaptation
*After the cardiac arrest it was mostly like this: Have I done everything before I fall asleep? Have I said good-bye to everybody…But after the last shocks it was not like that, I woke up in the night and jumped out of the bed* (laughter). *But now it is all right, now I don’t wake up in the middle of the night.*	Anxiety after cardiac arrest and inappropriate shocks. Finally coping.	ICD knowledge and worries	Hope
*Yes, it is after the shock it became worrisome…But I did not want to tell them* (husband and son)…*I think I keep a lot to myself. Sometimes it feels like I want to be alone…I don’t want them to call me ten times a day: how are you? Is everything ok?*	Anxious shortly after inappropriate shock. Does not share worries with close relatives.	Relationship and support	Adaptation
*One can’t just get too bogged down and worry. There are so many other things to be worried about. Damn, you have to live! That’s how I feel.*	Realistic view on risk of death. Accept risk.	Feeling healthy despite disease	Awareness
			Acceptance
*About friends’ concerns: I think they are ridiculous. But I say, oh God, what can I do about it? It’s over when it’s over…ha* (laughter).	Fatalistic view on death. Dissociates from friends worries.		Hope
Brother of a sudden death victim: *My mom became very worried about that time…but that is nothing we talked about very much. That is the way my family works…The ostrich method…it just buries the head in the sand and pretends like there is nothing.*	Sudden death affects family but they do not talk about it.	Relationship and support	Awareness
*I have such a bad background. They just dropped dead. On my mother’s side they just died, it started in the 40s…one was just 13 and the other 17…and I have a cousin…she was only 25…*	Aware of several cases of sudden cardiac death in the family.	Inheritance	Awareness

## Results

### Patient characteristics

Patient age ranged from 27 to 76 years (mean 57.7 years) and the majority (65.3%) was male. All New York Heart Association (NYHA) functional classes were represented. Time since the first known diagnosis of HCM varied (range 4–45 years, mean 16 years) Mean time with an ICD was 6.7 years and ranged from 2.4 to 16.6 years, with the exception of one patient who had an ICD for 1 year before explant due to heart transplant. Both primary (*n* = 20) and secondary ICD indications (*n* = 6) were represented. Experiences of at least one appropriate or inappropriate ICD shock were reported in 3 and 8 different patients, respectively. Characteristics of each participant are described in Table 1.

The findings of the study are presented as 10 narrative themes describing the experience of HCM patients living with an ICD. The order follows the narrative thread in the patients’ interviews and serves to depict that some things often happen before other. In the discussion section these narrative themes are further interpreted and reflected upon at a more abstract level in the light of 5 theoretical themes.

### HCM symptoms, diagnosis, and medication

Shortness of breath was the predominant HCM symptom, which was especially pronounced at exertion. Other symptoms were unspecific, such as tiredness, lack of stamina, syncope, and palpitations. Notably, no patient suffered from chest pain. ECG signs or a murmur sometimes lead to an echocardiography confirming HCM, but discovery also occurred during family screening or as a result of medical investigations for other reasons, including childbirth, general surgery, or when an infection, stroke, or cardiac arrest were managed.

The HCM diagnosis was often delayed and initially misdiagnosed as something else and the patients occasionally expressed worries about health providers´ actual knowledge about the disease. This trust was particularly damaged when a relative experienced SCD. Patients with a family history of SCD were easily convinced of the value of an ICD, whereas patients with other risk markers, such as non-sustained ventricular tachycardia sometimes questioned the need of an ICD before implant. At the time of interview, the term hypertrophic cardiomyopathy and its abbreviation HCM were unknown to many and they called the disease *an enlarged heart*, *heart trouble*, or *heart thickness*. One young patient said, *Then* (at the time of implant) *they said hypertrophic cardiomyopathy…and I really understood it*, while others required their physicians to write the term down for them. The patients reported high compliance with the prescribed HCM related medications (beta-blocker or calcium-channel antagonist) despite lack of short-term symptom relief, but dosage was often lowered due to presumed side effects.

### Inheritance

Although HCM is not always diagnosed early in life, women of childbearing age still reported that they wanted to have children despite the risks of passing on the condition to their children. One severely symptomatic older man said that he would have had fewer children if he had known what the disease progression would mean, but otherwise did not have much concern. Parents of young children pondered the consequences of genetic testing for their children. One couple talked openly to their child about HCM to avoid confusion. Even when patients received genetic counseling, they sometimes had only a vague understanding of how the disease can be passed on to their children. Cascade screening was challenging and sometimes impossible due to broken families, estrangements, and dysfunctional family dynamics, such as the young woman who could not be tested for HCM because her parents did not tell her that the disease ran in the family. No patient in the study blamed parents for their HCM.

### Implant decision, surgery, and wearing an ICD

Few primary prevention patients had a clear idea about the risk markers that made them eligible for ICD implant and sometimes these patients were not initially motivated to get an ICD. The experience of the implant procedure varied and they often recalled considerable pain. Complications requiring surgery were tolerated but some patients thought that preoperative information was sometimes lacking. Others reported feelings of isolation: *When I was waiting for surgery, I felt like a chicken going into the slaughterhouse*.

All young patients disliked people staring at the scars from the implant procedure, especially when bathing, but after some years many joked about the scar and claimed the ICD was a part of their body. An elderly woman said she initially avoided certain clothes which exposed the device, but later on, this did not trouble her. Some male patients even let people touch the scar. When ICD patients were playing with children, the device served as a reminder of the disease and made it real. A mother of a 5-year old daughter called it *the life-saver* and her daughter said she wanted one as well. Descriptions like *my heart runs on batteries* were common and show awareness and acceptance of living with technology. Gratitude, trust, and security were expressed along with a feeling of privilege because it is such a costly device. However, the device sometimes caused local irritation and required padding when using a seat belt or carrying a backpack; patients sometimes said they needed a cushion when lying in certain positions in bed.

### ICD knowledge and worries

A few patients knew that the ICD shock-function could temporarily be inhibited by magnet application; among patients who experienced inappropriate shocks this was common knowledge and some even had a magnet with them. Patients were worried about the lack of ICD-specific knowledge among health personnel. They had encountered this lack of knowledge in emergency care, primary care, and specialized care outside of cardiology units. The ICD card, which is provided to all patients, was considered helpful but there were suggestions for necklace or a bracelet with information, and one patient obtained one from a patient organization. Such easily visible identification could prove invaluable in an emergency situation, in which the patient was unable to communicate.

The difference between a pacemaker and an ICD was generally common knowledge among patients but they did not think this was known to the general public or among health care providers. The experience of the vibration alert function of some ICDs was sometimes confused with an ICD shock; some individuals realized this for the first time during their interview. The ICD device usually contraindicated medical investigations such as magnetic resonance imaging or transcutaneous electrical nerve stimulation which limited full access to health care. Some patients had reflected about deactivation in case of terminal illness and were concerned that health care providers would not recognize the ICD or distinguish it from a pacemaker at life’s end.

### ICD provides reassurance

All patients felt secure and grateful to receive an ICD. None regretted the decision to implant the ICD. They often had nicknames for the device, i.e. *my life-saver*, *the fire extinguisher*, *a friend of mine*, and *life insurance*. The word *secure* was announced numerous times by different patients. A young man whose father died suddenly at an early age felt overwhelmed and said, *Everybody should have one…I am protected but they are not….*

### ICD shocks

Even patients who experienced several inappropriate shocks persevered and accepted the therapy as part of their new life. Some of them came to terms with the shocks within a couple of weeks and one woman said, *You know that it* [the ICD] *actually works*. Other expressions were, *it was horrible, unpleasant, but I know I won’t die from it and I know how it feels…it is just a dreadful feeling*. Another commented, *It is damn nasty, really nasty, but there is no pain afterwards and it feels like a strong electrical discharge*. Typically they described their first feelings as, *scary, nasty, unpleasant, horrible, terrible, dreadful, and ghastly*. Metaphors for the shock were *being hit by a stone*, and *being shot by a revolver*, and *I jumped a foot, and I was like a jumping jack*. The unpredictable nature of shock therapy was described as a *bolt from out of the blue*, but these victims of inappropriate shocks came to terms with the shock and actually felt reassured after a few weeks. One patient who got the opportunity to talk to her device physician the same day a shock occurred, felt immediate relief. Most of the time, repeated ICD shocks caused a witness to summon an ambulance. Close relatives who witnessed a shock might become overly protective or avoid situations like the one that preceded the last shock. Although patients typically coped with the situation at the time it happened, relatives were also influenced by the dramatic event. A 4-year-old daughter avoided physical contact with her father for a short time after he experienced several shocks. One exceptional case involved a disappointed patient who had experienced complications, including device system infection and several inappropriate shocks due to a fractured Sprint Fidelis lead. She had considered (but rejected) device explant in favor of an external defibrillator. She described her situation as, *I can never relax … and be a human being again.* However, she appreciated the fact that her device had also delivered appropriate therapy. When experiencing an appropriate shock due to ventricular arrhythmia one patient typically fainted, but felt almost normal soon afterwards. It was a dramatic event for the people around him, but not for the patient who did not always seek medical attention after a shock. A survivor of a ventricular arrhythmia described his adrenergic response, *It was fantastic…I was sitting in a dark room and everything turned bright white*.

### Feeling healthy despite disease

Individuals spontaneously described themselves as *healthy* and did not perceive themselves as victims of disease. Their identity did not change even though they had to undertake several changes in their life. They typically denied being sick because they had adapted to a new lifestyle and accepted their limitations: *My husband has energy but I have almost no energy*, and *I learned to live with it*, and *I listen more to my body*… Upon reflection, they occasionally did realize that they had experienced a life-altering event. These changes may have kept them away from certain activities and was most dramatic after surviving a cardiac arrest. As time passed, patients coped with these events, reoriented themselves, and achieved new goals. When asked about his heart problem, an older patient replied: *Maybe I do not need a device. I feel healthy*. In patients with systolic heart failure or atrial fibrillation, physical limitations such as shortness of breath and tiredness were pronounced and they considered that it was HCM, not the ICD, that was severely limiting life. HCM affected their professional opportunities and made them dependent on other people’s help. Patients who had secondary prevention ICD felt safer and expressed gratitude that they had a new chance at life. For them, it was obvious that they had a severe disease but this did not make them give up their joy of living. Even though some secondary prevention patients admitted slight cognitive impairment, they still felt eager to continue their old activities. The family members of SCD survivors were often unable to continue ordinary life, expressed worries, and even suffered sleep disturbances according to the patients. In this group, it was common for partners to be overly protective of the ICD patient, especially when there were young children in the family. In general, young patients were more worried and felt more limited than older patients, who remembered the worries they had about their health during adolescence and early adulthood. A woman said, *My teenage years were difficult. I sometimes think: Why me? I am so nice, why couldn’t she get it instead? Still, I think everything is crap but I have become wiser and gained more perspective.* Shortly after diagnosis, patients usually had a feeling of being different but later accepted and adapted to the lifestyle changes their condition demanded, and did not see themselves as stigmatized.

### Leisure-time activities

In general, patients perceived their underlying HCM, rather than the ICD, as constituting the limit on their leisure-time activities. Athletic activities had to be avoided, modified, or restricted, and many patients were unsure about their recommended level of activity. Mountain trekking, badminton, ice hockey, soccer, dancing, swimming, and hunting sometimes had to be restricted, but patients adapted to these restrictions or switched to other activities. Young patients felt more limited by recommendations to restrict their activities than older patients did. In some cases, driving was restricted by the authorities but this restriction was not always communicated to the patient. In other instances, a concerned spouse might advise the patient against driving. Such advice was at times ignored. When the patient had to limit driving, this impacted both his leisure and professional activities. Physical intimacy was not affected by the device and patients did not express fear of shocks during sex. However presence of severe heart failure or comorbidity limited sexual performance. In fact, one patient who had intercourse the next day after ICD implant said, *We did it immediately when I came back from the hospital…just because I wanted to test it*.

### Professional life

Inability to work was associated with symptoms of HCM or comorbidities, especially atrial fibrillation and progression to heart failure or cognitive impairment in cardiac arrest survivors. Younger patients were more worried about their work and sometimes struggled to reorient themselves professionally. These adaptations included less travelling, avoiding stressful situations, reducing their workload, and accepting being on sick leave now and then. Sometimes colleagues helped with certain tasks such as climbing a ladder or heavy lifting. With age, the concerns about working capacity diminished. Looking back, patients with an early onset of symptoms had military service exemptions but thought they were otherwise free to pursue their own career goals. A welder had to change his line of work specifically because of the ICD and other patients sometimes could not pursue work that involved driving or electromagnetic exposure. If this brought on economic constraints, patients adapted to their modified standard of living and did not report it as being problematic.

### Relationship, support, and insurance

Following cardiac arrest, patients found their personal relationships were vastly changed. The survivor expressed gratitude, had a renewed appreciation for life, and modified goals and values. By contrast, the emotional response of family members was ambiguous…*The event has made us better connected but also creates problems…these worries can be really tiresome…on the other hand, we have a shared experience that somehow bonds us*. While patients were offered support, including referrals to psychology professionals from the health care system, family members were seldom involved. Generally, patients shared their feelings about their condition with the family, but occasionally the patients did not allow relatives to attend their clinical visits because they did not want them to worry, they expected it would result in overprotection and restrictions, or they wanted to make their own decisions independently but would introduce relatives later on. Occasionally (two patients) the disease was considered by the patient as a contributing factor in divorce. In younger patients, identification phenomena were observed, such as the man who became very anxious when he reached the age at which his father had died unexpectedly or when the child of an ICD patient said she wanted to get an ICD like her mother. In some cases when a patient and a family member went out for a walk, the family member would deliberately choose a less strenuous route to accommodate the patient. Having HCM caused emotional distress as well as physical symptoms in teenagers, who had an acute perception of themselves as being different from their peers.

No patient attended patient organization meetings, but a few had joined a Facebook group for ICD patients and one patient had found the American HCM patient association webpage. Older, highly symptomatic patients were less likely to use the internet as source of information than younger patients. Several patients expressed concern that they lacked information about their prognoses, which they considered the responsibility of their physicians to communicate to them. Not all of the information that patients found was considered beneficial. *The fact that athletes drop dead…it is not advantageous for me*. Furthermore, extensive talk and information about the disease sometimes conflicted with the patients’ self-image of being normal. Another young patient said, *The less you know, the less ill you are*. Swedish citizens are covered by a national insurance that pays for medical expenses, including the ICD. In addition, private insurance compensated certain patients for disability. Patients reported unexpected problems when renewing coverage or trying to sign up for a new policy.

## Discussion

**Fig. 1:**
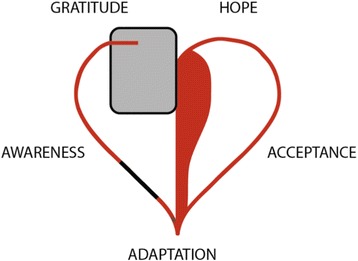
Theoretical themes emerging from narratives of HCM patients with ICD

Patients with ICDs due to HCM report various experiences and limitations throughout their lives, which is impacted by multiple external episodes in conjunction with their own personal traits. In the discussion, we want to introduce five theoretical themes, which we view as symbolic main threads in narratives presented in the result section. Despite the fact that these were individual perceptions, common theoretical themes emerged from them: awareness, adaptation, acceptance, gratitude, and hope (Fig. 1 and examples in Table 2). These themes were interpreted as influenced by the patients’ level of knowledge, support, and perceived limitations.

Even though HCM is the most common myocardial genetic disease, the awareness about HCM (even among health care providers) is often perceived as poor both among patients and their relatives [1, 10]. The lack of awareness about HCM and its terminology may be historical in nature, in that in the past, there have been >80 names to describe HCM [17] and its unspecific symptoms [1]. There is a lack of structured care for the HCM patient, who may ascribe this absence to ignorance of the disease. No patient had continued contact with any patient organization (there is currently no HCM specific support group in Sweden) and patients found social media irrelevant for communication about the disease. On the other hand, patients were grateful to the communications of their cardiologists, with whom they often had a long-term relationship. Patients acknowledged and appreciated the support they got from the cardiology clinic, although these clinics did not take a holistic approach and tended to limit themselves to device function and medical concerns. In particular, patients perceived that they did not get information about their prognoses, a finding supported by studies on general ICD patients [18–20]. Our patients did not expect emotional or existential support from health care systems, and turned to family instead. This could result in complicated situations. Patients sometimes reported that their family could be overprotective, imposing restrictions on them. On the other hand, family members could also be helpful, especially in doing physical tasks or coping with work situations, as previously described in general ICD populations [20, 21].

Many patients adapted, reduced their workload or even quit their jobs, which relieved stress and left time for them to enjoy family and friends but also limited social networking, leading to isolation and economic constraints [18]. This was more pronounced among younger patients in our study. This was described in a recent American study on HCM, in which investigators reported fear and identity changes among HCM patients [10]. In contrast, our patients adapted to their condition and accepted the limitations their disease put on them, but this did not change identity over their lifespan. Our patients were aware that their ICD offered protection from SCD and this resulted in gratitude and hope, which aligns with previous studies [22–25]. Before the ICD era, the health status of HCM patients assessed by questionnaires, was significantly lower than normal population [26]. While overall disease management has likely improved the situation, the authors feel that the ICD may also relieve some of the stress as it protects against potentially life-threatening SCD. Both primary and secondary indication patients appreciated their lives and considered the device a valuable life-saver contained within their body. It was accepted but constituted restriction and adaptation with regard to specific activities. The ICD was neither a reminder of death nor a cause for anxiety. This reassurance was also valid even after a history of ICD shocks (both appropriate and inappropriate). The time period after ICD shock for regaining calmness and acceptance was typically a few weeks, which is shorter than cross-sectional and longitudinal findings from other ICD populations but consistent with an actual time dependent improvement [19]. Patients with inappropriate shocks recalled an unpredictable, unpleasant episode that often led to urgent hospital visits, whereas appropriate shocks were not perceived as painful, probably because the patient received therapy while unconscious. Inappropriate shocks are often multiple, and are the major drawback of ICD therapy. Efforts are warranted to reduce this risk, especially in a HCM population with extended life expectancy [27]. However, even in the case of inappropriate shocks, our patients were reassured, accepted, and understood the benefits of the life-saving ICD. No patient in our study regretted having the ICD implanted. We believe that in addition to careful information about risk of shocks, patients should be given balanced, not exaggerated, information. Even though receiving an ICD shock is unpleasant and temporarily distressing, patients were surprised that it was not as bad as they expected it to be. Our results indicate that shock therapy affects the whole family and the spouse’s response to therapy should not be ignored or trivialized [21, 24].

Patients thought that their everyday activities were restricted by HCM and comorbidity, rather than the ICD. This adaptation is supported by previous findings as in the general ICD populations [28, 29]. Overall, patients were able to adapt and accept obstacles over the course of their lives. Patients with HCM and ICDs encountered challenges, but still had a strong life spirit, great hope, and accepted the lifestyle adaptations they had to make, but were grateful to their device as a life-saver. HCM is a heterogeneous disease and only a subset of HCM patients are indicated for ICD therapy. Nevertheless, it is important to recognize in this diverse patient population that patients can still enjoy their lives, experience joy and hope, learn new things, and have meaningful communications and interactions with others.

### Study strengths and weaknesses

This study focuses on ICD recipients specifically due to HCM, in a large cohort. We identified eligible patients from the validated Swedish ICD Registry which covers all implants [15]. The diagnosis of HCM was validated through reading of medical records to ensure correctness of register data on ICD indication. All patients consented and participated in the interviews from the two regions which reduces selection and referral center bias. All patients were fluent in Swedish and were able to express themselves well. The interview guide fulfilled the purpose of covering different topics and was useful to catalyze elaborate and rich narratives. One of the study’s strengths is the rich data, both in quantity and quality. The amount of data was also a challenge. Here the combination of latent content analysis to condense and bring order to the data combined with hermeneutics to analyze and interpret data was useful. The research team continuously reflected on the pre-understanding trough the discussions and repetitive readings of the texts to ensure that interpretations were grounded in data. Notably, self-reported experiences may differ from a relative’s views and longitudinal experiences recalled by patients may be different than findings from studies using follow-up interviews. The explorative design is beneficial because it gives insight in a new field but findings need to be confirmed in further confirmative studies. However, the generalizability to other geographical areas and cultural contexts needs to be addressed in future studies.

## Conclusions

HCM patients with ICDs perceive that their poor health is the result of the burden of HCM symptoms, especially shortness of breath at exertion. The slow progression of HCM allows patients to adapt to the disease and accept limitations it may impose. HCM patients feel hope and reassurance for the future despite their disease state. To some extent, patients reprioritize their lives from professional activities to value family from whom they seek support. Support is usually obtained from the family rather than health care professionals, whom they consider as mostly a technical service of disease management. They feel grateful to the life-saving ICD and they trust the device and consider it as an integral part of their body, which contributes to hope as they continue with their lives. Inappropriate as well as appropriate shocks may result in temporary concerns but patients usually cope with them (adapt and accept) and rationalize them within a short period of time. ICD treatment is well tolerated among HCM patients but knowledge about it varies substantially. This emphasizes the importance of raising awareness about HCM and increasing knowledge about the role of ICD therapy and tailored disease management in the care of HCM patients. Improvement of clinical care should facilitate awareness, adaptation, and acceptance during the patients’ life course. This approach will give hope when encountering challenges.

## Appendix 1

**Table 3:** The interview guide is a framework of the areas relevant to the exploration of the life experiences of HCM patients with ICDs. It should be considered a support tool for conducting the interview using an open question format

Background	How old are you?
	Do you live with anybody?
	Do you have children?
Early questions	What is it like to live with HCM and ICD?
	How and when did you get the HCM diagnosis?
	When did you get the ICD?
General health	What do think about your health?
	How do other people consider your health?
	In what way does the ICD affect your health?
	Has your health changed over time?
	What do think about your future health?
Professional life	Are you working/studying?
	Has your professional life been affected by HCM/ICD?
	Do you think your future career will be affected by HCM/ICD?
Leisure time	In what way has you leisure-time been affected?
	Do you exercise? How does that work?
	Did you get advice on activity levels? Do you follow this advice?
Family & Friends	Is family life affected by HCM/ICD?
	How did your relatives know about your HCM diagnosis and ICD?
	What do your family and close friends think about your having an ICD?
	What do your family and friends know about your HCM and ICD?
Driving	Do you have a driver’s license? Which certificates?
	Is your driver’s licenses affected by HCM/ICD?
	Did you drive for a living?
	What advice did you get about driving?
Insurance	Did your insurance company act differently based on your HCM/ICD?
Lifestyle	What kind of food do you eat? Alcohol? Smoking?
Medication	Which pharmaceutical drugs do you take?
	Do you take these prescribed drugs?
	Do think that these drugs relieve symptoms/cause side effects?
Diagnosis of HCM	What made them suspect HCM? How long did it take to be diagnosed?
ICD	When and why did they decide about the ICD?
	What do you think about the information before ICD implant?
	Did you experience ICD shock (appropriate/inappropriate)?
	How was the implant procedure?
	Did you experience any surgical complications?
	Does the ICD give you a sense of security?
	Did you ever regret receiving an ICD?
	Do you know how to turn the ICD off?
	What is the difference between a pacemaker and an ICD?
Health care	What could be improved in health care in HCM and ICD?
	Did you ever contact a patient association?
	Do you use internet/social media? For HCM/ICD communication?
	What can the society do to improve care for HCM/ICD patients?
Sexuality	Is your sex life affected by HCM/ICD?
	Did you need medication to improve sexual performance?
Reproduction	What are your concerns about your child getting HCM?
Pregnancy	Did HCM/ICD affect your pregnancy?
Genetics	Have they found a mutation causing your HCM? Did the genetic counselling affect the family?
Sleep	How is your sleep quality?
	Has you sleep been affected by HCM/ICD?

## Abbreviations

HCM: Hypertrophic cardiomyopathy
ICD: Implantable cardioverter defibrillator
NYHA: New York Heart Association
SCD: Sudden cardiac death
